# Supplementary material for: Function of BriC peptide in the pneumococcal competence and virulence portfolio
Source: PLoS Pathog. 2018 Oct 11;14(10):e1007328. doi: 10.1371/journal.ppat.1007328 (PMC6181422; doi:10.1371/journal.ppat.1007328)
Supplement: S1 Result — (DOCX) [file ppat.1007328.s001.docx]

**Supplementary Result**

**Examining the role of *briC* in transformation**

One of the main phenotypic consequences of the competence pathway is transformation. Since ComE regulates the expression of *briC*, we investigated whether *briC* plays a role in regulating transformation efficiency. To this end, we added different amounts (100ng or 500ng) of exogenous DNA (genomic or amplified linear fragments) to pneumococcal cells. We found a minor decrease in the transformation efficiency of the *briC-*deletion mutant cells (R6DΔ*briC*) relative to the WT cells (**Fig. S1**). A strain with complemented *briC* (R6DΔ*briC*::*briC*) exhibited a partial restoration of this defect. A strain with overexpression of *briC* (R6DΔ*briC*::*briC*-OE) displayed a greater restoration of the transformation efficiency, as compared to R6DΔ*briC*::*briC* cells, but not a full rescue (**Fig. S1B**). These results suggest that *briC* may play a role in regulating transformation efficiency.
