# Supplementary material for: Function of BriC peptide in the pneumococcal competence and virulence portfolio
Source: PLoS Pathog. 2018 Oct 11;14(10):e1007328. doi: 10.1371/journal.ppat.1007328 (PMC6181422; doi:10.1371/journal.ppat.1007328)
Supplement: S1 Fig — (A) Transformation efficiency for WT, ΔbriC and ΔbriC::briC cells in strain R6D using exogenous (i) genomic DNA, and (ii) linear DNA. X-axis denotes amount of exogenous DNA added in cells. (B) Transformation efficiency for WT, ΔbriC, ΔbriC::briC, and ΔbriC::briC-OE cells in strain R6D using 500ng of exogenous linear DNA. Y-axis denotes the fold change in transformation efficiency relative to WT cells. Cells were grown in Columbia broth at pH 6.6 to an OD600 of 0.05, and were treated with CSP1 along with specR exogenous DNA for 30 minutes followed by plating on Columbia agar plates supplemented with spectinomycin (100μg/ml). No colonies were observed for samples treated with exogenous DNA in the absence of CSP1. Error bars represent standard error of the mean calculated for at least three biological replicates; ‘ns’ denotes not significant,* p<0.05, *** p<0.001, **** p<0.0001 using ANOVA followed by Tukey’s post-test. (PPTX) [file ppat.1007328.s002.pptx]

## Slide 1
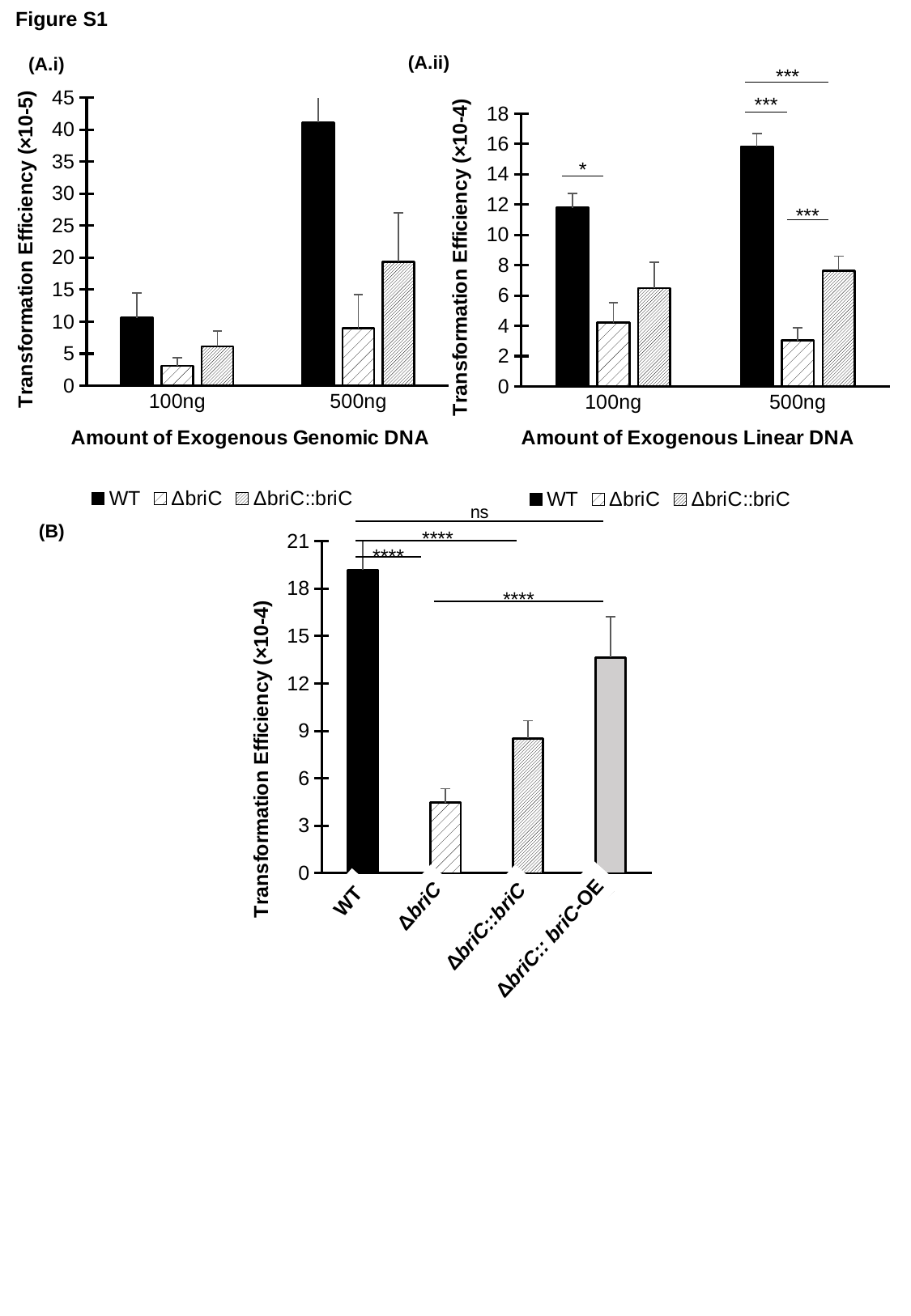

Figure S1
(A.ii)
(A.i)
***
### Chart
| Category | WT | ΔbriC | ΔbriC::briC |
|---|---|---|---|
| 100ng | 10.61637346883451 | 3.104827331573655 | 6.120639049045145 |
| 500ng | 41.10263136648921 | 8.96278987556561 | 19.35830476101864 |***
### Chart
| Category | WT | ΔbriC | ΔbriC::briC |
|---|---|---|---|
| 100ng | 11.78795154661475 | 4.212751380584611 | 6.491048068507085 |
| 500ng | 15.82864662196265 | 3.025121713370653 | 7.644965462588405 |*
***
ns
(B)
****
### Chart
| Category | Av |
|---|---|
| WT | 19.1774869233519 |
| ΔbriC | 4.463265720814018 |
| ΔbriC::briC | 8.5209864227277 |
| ΔbriC::briC-OE | 13.64793331668332 |****
****
WT
ΔbriC
ΔbriC::briC
ΔbriC:: briC-OE
