# Supplementary material for: Function of BriC peptide in the pneumococcal competence and virulence portfolio
Source: PLoS Pathog. 2018 Oct 11;14(10):e1007328. doi: 10.1371/journal.ppat.1007328 (PMC6181422; doi:10.1371/journal.ppat.1007328)
Supplement: S2 File — (DOC) [file ppat.1007328.s010.doc]

>P*briC*

Agaatgactaaatgagactagaaaggtctcattttttatggaaaaatgcaattcaggtcg

aaaaattgccagttgaggagatagactataaaatagacaaaaaaagaaatatactataaa

tgtgagcctacaacaatctattttaagaaggtacttgca

>P*briC*_long_

agaatgactaaatgagactagaaaggtctcattttttatggaaaaatgcaattcaggttg

aaaacttgacagttgaggagatagactataaaatagacaaaaaagatgtatactgtagat

gtaagcttacaaaaatttatagtgtattgaatctataacagtacaccttgactgctaaaa

tatttctataaattaatttgactttcctgatagagttgtttacatcttatttcaaatcac

tatattttaagaaggtacttgca

>P*briC*_long_ (with RUP sequence in bold)

agaatgactaaatgagactagaaaggtctcattttttatggaaaaatgcaattcaggttg

aaaacttgacagttgaggagatagactataaaatagacaaaaaagatgtatactgtagat

gtaagcttacaaaaatt**tatagtgtattgaatctataacagtacaccttgactgctaaaa**

**tatttctataaattaatttgactttcctgatagagttgtttacatcttatttcaaatcac**

**tata**ttttaagaaggtacttgca
